# Intratumoral regulatory T cells from colon cancer patients comprise several activated effector populations

**DOI:** 10.1186/s12865-021-00449-1

**Published:** 2021-08-19

**Authors:** Louis Szeponik, Filip Ahlmanner, Patrik Sundström, William Rodin, Bengt Gustavsson, Elinor Bexe Lindskog, Yvonne Wettergren, Marianne Quiding-Järbrink

**Affiliations:** 1 Department of Microbiology and Immunology, Institute of Biomedicine, The Sahlgrenska Academy at the University of Gothenburg, Box 435, 405 30 Göteborg, Sweden; 2 Department of Surgery, Institute of Clinical Sciences, The Sahlgrenska Academy at the University of Gothenburg, Blå stråket 5, 413 45 Göteborg, Sweden

**Keywords:** Regulatory T cell, Colon cancer, Cancer-specific survival, CD39

## Abstract

**Background:**

Intratumoral regulatory T cells (Treg) in colon cancer are a heterogeneous cell population, with potential impact on patient outcome. Generally, a high Treg infiltration has been correlated to a worse patient outcome, but it is still unclear how the composition of different Treg subsets affects patient relapse and survival. In this study, we used mass and flow cytometry to characterize Treg in colon tumors and corresponding unaffected tissue, followed by a correlation to clinical parameters and patient outcome.

**Results:**

Using mass cytometry, we defined 13 clusters of intestinal Treg, three of which were enriched in the tumors. The two most enriched clusters were defined by their expression of the proliferation marker Ki67 and CD56, respectively. The Treg accumulating in the tumors expressed inducible T-cell co-stimulator (ICOS), OX-40, and CD39, indicating that they were effector Treg (eTreg). Intratumoral CD39^+^ Treg also had a higher expression of Foxp3, suggesting a higher suppressive activity, and we subsequently used CD39 as a marker for eTreg. Our further studies showed that colon tumors can be divided into two tumor groups, based on the proportion of CD39^+^ putative eTreg in the tumors. This property was independent of both tumor microsatellite status and tumor stage, which are important factors in predicting cancer disease progression. In a prospective study of forty-four colon cancer patients, we also showed that patients with a high CD39 expression on tumor-infiltrating Treg have a tendency towards a less favorable patient outcome in terms of cumulative cancer-specific survival.

**Conclusions:**

This study uncovers novel subsets of tumor-infiltrating Treg in colon cancer, and suggests that CD39 may be a potential therapeutic target in patients with microsatellite stable colon tumors, which are usually refractory to checkpoint blockade therapy.

**Supplementary Information:**

The online version contains supplementary material available at 10.1186/s12865-021-00449-1.

## Background

In recent years, it has become evident that the immune system can recognize and sometimes efficiently clear transformed neoplastic cells [1]. One example of this is colorectal cancer (CRC) where lymphocyte infiltration into tumors clearly correlates to patient outcome [2–4]. In this context, presence of CD8^+^ memory cells and Th1 cells improve patient prognosis, while alternatively activated macrophages and Th17 cells correlate with a worse prognosis. Indeed, the immunoscore, a scoring system that quantifies tumor-infiltrating immune cells, is a more accurate method than tumor staging to predict patient outcome in CRC [5]. Cancer immunotherapies, in particular immune checkpoint blockade, are now established targeted therapies, which shift the balance between immunostimulating and immunosuppressive forces to the benefit of the patient [6]. Immune checkpoint blockade has had considerable effect in the treatment of several solid cancer forms, e.g. lung cancer, advanced malignant melanoma and renal cell cancer, but a favorable effect in colon cancer has only been shown for microsatellite instability high (MSI-H) tumors, while microsatellite stable (MSS) tumors show poor response [7, 8]. MSI-H tumors have a deficient DNA repair machinery and a subsequent increase in mutational load, creating an immunogenic tumor environment and higher lymphocyte infiltration [9].

In addition to continued efforts in improving immune checkpoint blockade there is a need for alternative strategies for immunotherapy. One target for such treatment is regulatory T cells (Treg), which are accumulating in most solid tumors and are associated with a poor patient prognosis [10]. Initially, several studies in colorectal cancer patients showed a correlation between tumor-infiltrating Foxp3^+^ putative Treg cells and a favorable prognosis [11–14]. However, a more recent study by Saito et al*.* showed that a high infiltration of carefully identified Treg in the tumor actually correlates to a poor prognosis in CRC [15], and De Simone et al*.* also correlate intratumoral Treg numbers with a less favorable patient outcome in CRC [16]. It is clear from functional studies that tumor-infiltrating Treg suppress conventional T cell proliferation and cytokine production [15–18]. Several studies also indicate that they may actually be more suppressive than Treg found in the unaffected colon mucosa, based on expression of immunoregulatory surface receptors or suppression of effector cell proliferation [15, 16, 18, 19]. Thus, the contribution of tumor-infiltrating Treg to patient prognosis in colorectal cancer is still not completely defined, but it would appear that Treg infiltration into established tumors promotes tumor progression.

Considering that Treg in cancer patients would probably have to be targeted on a subpopulation basis due to extensive autoimmune side-effects when targeting all Treg [20], the functional differences between intratumoral Treg subsets needs to be investigated. To better define intratumoral Treg, we performed mass cytometry analyses of Treg from tumors and unaffected mucosa from colon cancer patients. We identified three Treg clusters that accumulate in colon tumors, which were distinguished by their CD39 expression, and two that were reduced in the tumors. Subsequent flow cytometry analyses showed that colon tumors could be divided into two types, one with a high expression of CD39 by Treg and one with an intermediate expression. In patient outcome analyses, patients with a high CD39 expression by tumor-infiltrating Treg had a worse outcome.

## Results

### Mass cytometry identifies 13 clusters of intestinal Treg in colon cancer patients

**Fig. 1:**
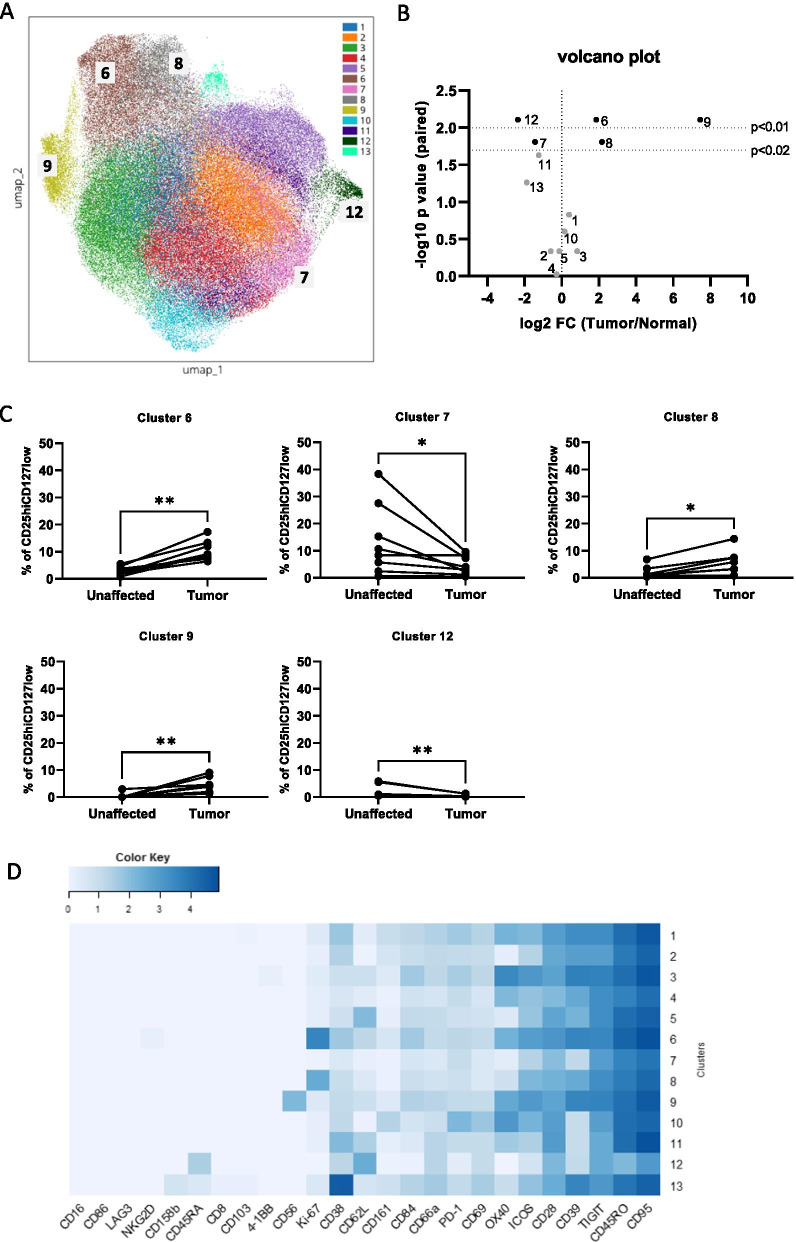
Unsupervised clustering of Treg from colon tissues. Single CD45^+^ cell suspensions from unaffected colon tissue and tumors were analysed by mass cytometry. **A** Phenograph clustering of Treg resulted in 13 metaclusters which are overlayed on the corresponding UMAP plot. **B** Volcano plot shows the fold change of the median cluster frequencies plotted against the *p* value of the paired analysis between unaffected and tumor tissue. **C** Frequencies of cells designated to the significantly increased or decreased clusters in unaffected and tumor tissue. Symbols represent individual values and values from the same patient are connected. **D** Heatmap showing the median signal intensities of the respective markers in the different clusters. Color coding is applied over the whole heatmap. *p < 0.02, **p < 0.01, n = 8

To obtain an unbiased overview of Treg subpopulations in colon tumor tissues, the CD45^+^ fraction of mucosal single cell suspensions from both unaffected colon tissue and tumors from 8 consecutive patients (Additional file 1: Table S1) were analysed by mass cytometry. As previously demonstrated [15, 21, 22], Treg frequencies were increased in the tumors compared to unaffected tissue (1.9 ± 1.3% Treg among all CD3^+^ cells in unaffected tissue, and 5.6 ± 3.0% in tumors), and the intraepithelial lymphocyte (iEL) fractions contained virtually no Treg. Treg were manually gated (Additional file 1: Figure S1) before unsupervised clustering and dimensionality reduction was performed. We identified 13 mucosal Treg metaclusters in the lamina propria lymphocyte (LPL) fractions across multiple patients and the two tissue types (Fig. 1A). Originally, 18 clusters were computed, but 5 clusters contained events less than 1% of all Treg in both tumors and unaffected tissues and were excluded from further analysis. Metacluster distribution was then identified among the two tissue types and the patients for further analysis.

Of the 13 clusters identified in the LPL fraction, two (cluster 6 and 9) were significantly increased in the tumors at *p* < 0.01 and one (cluster 8) at *p* < 0.02 (Fig. 1B). The three enriched clusters grouped together at one end of the UMAP plot, indicating similarity (Fig. 1A). Cluster 6 was one of the more numerous in the tumors, comprising on average 10.3% of intratumoral Treg, and was increased 3.6-fold compared to unaffected colon. Cluster 9 made up an average of 4.6% of tumor-infiltrating Treg, but was increased from virtually no cells at all in the unaffected tissue, resulting in a median 175-fold increase (Fig. 1B, C). Cluster 6 cells appear to correspond to activated effector/memory Treg based on their expression of CD45RO, inducible T-cell co-stimulator (ICOS; CD278), OX-40 (CD134), and CD39. Cluster 8 has a similar expression of CD45RO, ICOS, and CD39, and the major difference between cluster 6 and 8 is the relatively low expression of OX-40 in cluster 8 (Additional file 1: Figures S2 and S3). Both cluster 6 and 8 comprise proliferating cells, as judged by Ki67 expression, which is virtually absent in all other clusters (Fig. 1D). The highly enriched cluster 9 also comprises CD45RO^+^ICOS^+^OX-40^+^CD39^+^ cells but is distinguished from the other clusters by a high CD56 expression (Fig. 1D, Additional file 1: Figures S2 and S3).

Furthermore, two clusters were significantly reduced in the tumors compared to the unaffected mucosa (cluster 7 and 12, *p* < 0.02 and *p* < 0.01, respectively; Fig. 1B). They both comprise cells with a low expression of ICOS, OX-40, and CD39. Cluster 12 is relatively small, and has a lower expression of CD45RO and higher CD45RA and L-selectin (CD62L) expression than any other cluster, and might thus represent naïve Treg (Fig. 1D). Cluster 7 is one of the most numerous in the unaffected tissue, and has a lower expression of both the activation and exhaustion markers assessed in the current panel, and may correspond to resting memory Treg. Additional file 1: Figure S4 shows frequencies of the remaining clusters in unaffected and tumor tissue.

Virtually all the Treg in the tissues express the effector molecule CD95 (Fas) and the immune checkpoint receptor T cell immunoreceptor with Ig and ITIM domains (TIGIT), while another immune checkpoint, lymphocyte activation gene 3 (LAG-3), has a limited expression in all clusters. The best characterized immune checkpoint marker, programmed cell death protein 1 (PD-1; CD279), has an intermediate expression, but is not differentially expressed in any of the enriched clusters (Fig. 1D, Additional file 1: Figure S2). When comparing the clusters that were either increased or decreased in the tumors, the best markers to separate them, among those used in the current study, are ICOS, OX-40, and CD39 (Additional file 1: Figure S3). ICOS and OX-40 are co-stimulatory molecules expressed on activated cells, while CD39, an ectoenzyme converting ATP to AMP as a first step to generate immunosuppressive adenosine [23], marks effector Treg (eTreg) with improved suppressive ability [24–26]. The expression of CD39 also correlates well with the enriched clusters 6, 8, and 9, and is lower in cluster 7 and 12 (Additional file 1: Figures S2 and S3).

### The frequency of CD39^+^ intratumoral Treg divides patients into two groups

**Fig. 2:**
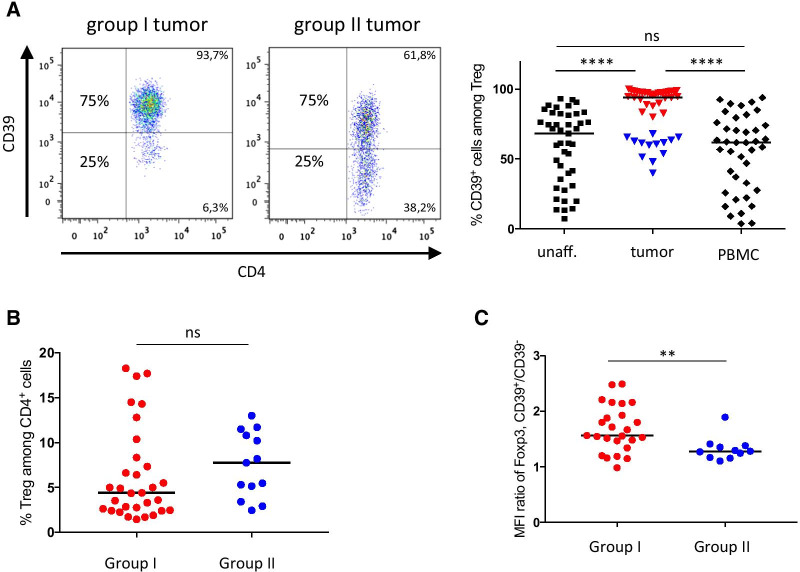
CD39 expression by Treg from colon adenocarcinoma patients. Frequencies of CD39^+^ Treg among total Treg, were determined by flow cytometry in single cell suspensions from tumors, unaffected colon tissue and peripheral blood. Colon tumors were divided into two groups based on CD39 expression by isolated intratumoral Treg, i.e. group I tumors (≥ 75% of CD39-expressing cells among intratumoral Treg) and group II tumors (< 75% of CD39-expressing cells among intratumoral Treg). **A** Representative FACS-plots, depicting CD39 expression by Treg from one group I tumor and one group II tumor, and a compilation of individual data points from the different locations (*n* = 40–44), with tumor-infiltrating Treg color-coded with red (group I) and blue (group II). **B** Lymphocytes isolated from group I and II tumors were analyzed for frequencies of total Treg among intratumoral CD4^+^ T cells. **C** MFI of Foxp3 expression (*n* = 36), shown as the MFI ratio of Foxp3 expression, between CD39^+^ and CD39^−^ Treg in patients belonging to group I and II. Symbols represent individual values and horizontal lines the median. Individual values are color-coded in red (group I) and blue (group II). **p < 0.01, ****p < 0.0001

As CD39 could be used as a proxy marker for the subsets of activated Treg accumulating in colon tumors, we extended our studies to a set of 44 consecutive patients, and used flow cytometry to identify CD39^+^ Treg in tumors, unaffected tissue, and blood (see Additional file 1: Figure S5 for gating strategy). These analyses showed that patients could be divided into two well-defined groups based on the frequency of CD39 expressing cells among intratumoral Treg (Fig. 2A), denoted here as group I who have tumors with ≥ 75% of CD39-expressing cells among intratumoral Treg and group II with < 75% of CD39^+^ Treg. In contrast, no clear division into groups could be seen when analysing circulating Treg and Treg from unaffected colon tissue in our patient cohort. It should be noted that there was no significant difference between group I and II patients in total intratumoral Treg frequencies (Fig. 2B). We also compared frequencies of CD39^+^ Treg between tissues to investigate if circulating Treg may give an indication of CD39 expression by intratumoral Treg. Indeed, when both patient groups were analyzed together, it was clear that CD39 expression among Treg correlated positively and significantly between all tissues (*p* < 0.001; Additional file 1: Figure S6). Intratumoral Treg had a near universal expression of CD39 when frequencies of CD39-expressing Treg reached 60% in peripheral blood or unaffected colon tissue.

It was previously shown that Treg effector cells are all found in the Foxp3^high^ population [14]. We thus examined Foxp3 expression in the tumor-infiltrating Treg to correlate with CD39 expression. These analyses showed that the Foxp3 mean fluorescence intensity (MFI) ratio between CD39^+^ and CD39^−^ intratumoral Treg was greater in colon tumors of patients from group I, with high CD39 expression, compared to Treg from group II with low CD39 expression (Fig. 2C). Taken together, these findings suggest that colon cancer patients may be divided into two groups based on the frequency of CD39^+^ intratumoral Treg and that patients with a high frequency of CD39-expressing Treg in the tumor may also have larger numbers of effector Treg, with a more activated phenotype driven by high Foxp3 expression.

### CD39^+^ Treg frequencies do not correlate to clinical parameters

**Fig. 3:**
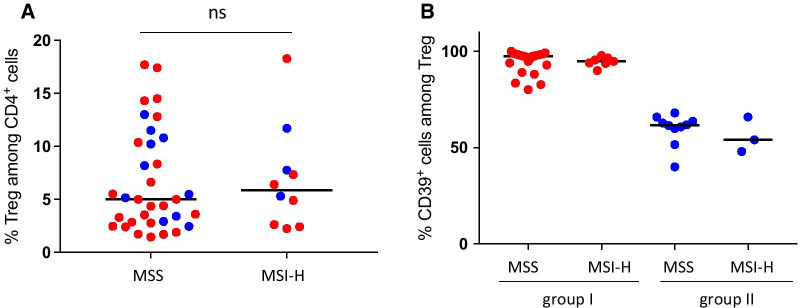
Frequencies of intratumoral Treg and CD39^+^ Treg in relation to tumor microsatellite status. Frequencies of CD39^+^ Treg were determined by flow cytometry in cell suspensions isolated from colon tumors. **A** Frequencies of Treg among intratumoral CD4^+^ cells in MSS tumors and MSI-H tumors (*n* = 44). **B** Frequencies of CD39^+^ Treg among intratumoral Treg in MSS tumor and MSI-H tumors of patients belonging to group I and II (*n* = 44). Symbols represent individual values and horizontal lines the median. Individual values are color-coded in red (group I) and blue (group II)

The microsatellite status and tumor stage of colon tumors are important factors determining the ensuing immune response to the tumor as well as patient outcome in colon cancer [2, 27, 28], and we next wanted to investigate frequencies of CD39^+^ putative eTreg in the tumors in relation to clinical parameters. All MSI tumors included in the study, were characterized as MSI-H, and no MSI-L tumors were detected. We observed similar frequencies of total intratumoral Treg among CD4^+^ T cells in MSI-H tumors compared to MSS tumors (Fig. 3A). To investigate if MSS/MSI-status in colon cancer could contribute to the presence of CD39^+^ intratumoral Treg, we stratified our sample for group I and II, but there was no significant difference between MSI-H and MSS tumors regarding the frequency of intratumoral CD39^+^ Treg (Fig. 3B).

**Fig. 4:**
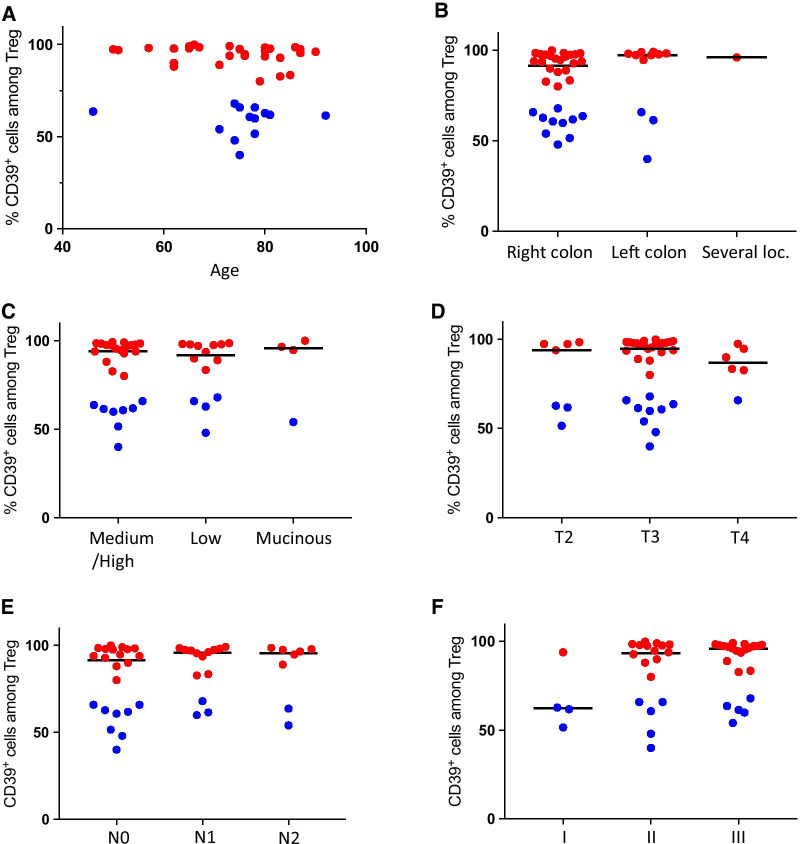
Frequencies of intratumoral CD39^+^ Treg in relation to clinical parameters. Frequencies of CD39^+^ Treg were determined by flow cytometry in cell suspensions isolated from colon tumors. Frequencies of CD39^+^ Treg among intratumoral Treg in relation to age (**A**), tumor localization (**B**), tumor differentiation grade (**C**), T stage (**D**), lymph node spread (**E**), and tumor stage (**F**). Symbols represent individual values and horizontal lines the median. Individual values are color-coded in red (group I) and blue (group II)

Additionally, we analyzed CD39 expression by intratumoral Treg in relation to prognostically important clinical parameters such as age, tumor localization, tumor differentiation grade, T stage, lymph node spread and tumor stage (Fig. 4). Group II patients were, except for two patients, clustered in the age span between 71 and 78, but apart from this observation, there was no correlation between age and CD39 expression by intratumoral Treg (Fig. 4A). In addition, there was no clear relationship between CD39 expression by intratumoral Treg or tumor location (Fig. 4B). Furthermore, CD39 expression by tumor-infiltrating Treg did not differ significantly between tumors with varying differentiation grade, different T stage or lymph node spread, and neither with tumor stage (Fig. 4C–F). We could also note that patients in group I or group II did not accumulate in any of the subgroups divided according to tumor characteristics (Fig. 4B–F). We conclude that, at the stage of primary surgery, the presence of CD39^+^ intratumoral eTreg is likely not dependent on established prognostically important clinical parameters, such as tumor stage or microsatellite status.

### High levels of CD39 expression among intratumoral Treg may correlate to a worse patient outcome

**Fig. 5:**
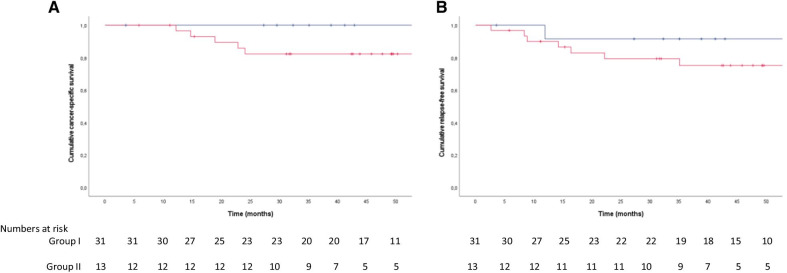
Frequencies of intratumoral CD39^+^ Treg and patient outcome. Preliminary data on cumulative cancer-specific survival (**A**) and cumulative relapse-free survival (**B**), of group I patients (≥ 75% of CD39-expressing Treg) and group II (< 75% of CD39-expressing Treg). Kaplan–Meier curves are color-coded for patients belonging to group I (*n* = 31; red) and II (*n* = 13; blue) and the number at risk in each group at specified time-points is depicted below each graph. Small vertical bars on the Kaplan–Meier curves indicate censoring

In light of the recent studies correlating high Treg infiltration in colon tumors with a poor patient outcome [15, 16], we compared the prognosis for patients with high and low CD39 expression, to investigate if the assumed functional difference between the CD39^+^ and CD39^−^ intratumoral Treg subsets in colon cancer may affect patient outcome. Using survival analysis and comparing Kaplan–Meier curves for group I and group II during a follow-up period of at least three years, we observed a tendency towards higher cumulative cancer-specific survival in group II, with a lower proportion of CD39^+^ Treg compared to group I, which was not statistically significant (*p* = 0.131; Fig. 5A). However, the cumulative relapse-free survival did not differ to the same extent between the groups (Fig. 5B). Due to the low frequency of events, we could not perform multivariate analyses. However, the absence of correlation between intratumoral CD39^+^ Treg frequencies and both microsatellite status and clinical parameters, may suggest that CD39 expression by Treg is a relatively independent survival predictor. Taken together, survival data suggests that patient prognosis in colon cancer may be affected by the degree of intratumoral accumulation of Treg with an activated phenotype, pointing towards the possibility that accumulation of functionally distinct Treg subsets in the tumor microenvironment may affect patient outcome.

## Discussion

In this study, we present a detailed examination of Treg phenotypes in colon tumors and preliminary survival data for colon cancer patients, which suggests that a large intratumoral accumulation of eTreg, defined by their expression of CD39, may correlate to a poor patient prognosis.

It is well known that Treg accumulate in solid tumors, and here we provide an unbiased multiparameter assessment of the specific Treg populations that are infiltrating human colon tumors. Based on suppression assays conducted in vitro, tumor-infiltrating Treg in colorectal cancer can exert immunosuppressive functions via programmed death-ligand 1 (PD-L1), IL-10 and TGF-β [16, 19]. In addition, tumor-infiltrating Treg will probably also act to suppress surrounding effector immune cells through the activity of CD39 and ultimately adenosine signaling, similar to what is suggested for intratumoral Treg in breast cancer patients based on in vitro experiments [29].

A striking feature of the enriched Treg populations detected in our study is their activated phenotype with expression of the co-stimulatory molecules ICOS and OX-40, combined with the effector molecule CD39. There is also an almost uniform expression of the exhaustion marker TIGIT among the tumor-infiltrating Treg, while other immune checkpoint receptors associated with exhaustion (PD-1 and LAG3) were not expressed to any larger extent in the enriched clusters. This is somewhat surprising, but might indicate that the Treg accumulating in colon tumors represent activated eTreg that are not yet under the influence of immunomodulatory checkpoint signaling. The ongoing proliferation in two clusters and the generally high expression of activation markers in the enriched clusters would also support this conclusion. Unfortunately, the cell numbers that can be retrieved from tumor tissue only occasionally allows for cell sorting and functional assays. The Treg accumulating in tumors were also low in CD103, which has been used as a marker of tissue resident memory T cells. However, recent findings indicate that this is primarily true for CD8^+^, but not CD4^+^, T cells in the human intestinal mucosa [30]. We could also identify one enriched cluster which is distinguished by a strong expression of CD56 (cluster 9). This may seem contra-intuitive, as CD56 is mainly expressed by NK cells and by unconventional T cells. However, a recent study identified high frequencies of TGF-β-producing CD56^+^ Treg in active lesions from Langerhans cell histiocytosis [31]. Suppressive CD56^+^ Treg have also been detected in hepatocellular carcinoma [32]. These authors speculated that the tumor microenvironment may convert infiltrating CD56^+^ T cells into functional Treg, possibly by TGF-β, but if this is the case in CRC remains to be examined. Taken together, the common feature of all the enriched Treg clusters in the tumors is a strong expression of ICOS, OX-40, and CD39, which are not seen in the contracting clusters. Of these markers, CD39 is a well-established effector molecule of potent Treg, and correlates well to an activated phenotype [17, 18, 24, 25].

CD39 is also known as ectonucleoside triphosphate diphosphohydrolase-1 (ENTPD1), an ectoenzyme catalyzing the first and rate-limiting step in the conversion of extracellular ATP to immunosuppressive adenosine, which is one of the main effector functions in Treg [24, 33, 34]. A further consequence of this enzymatic activity is the removal of pro-inflammatory extracellular ATP, which activates myeloid cells through the NLRP3 inflammasome. Adenosine may act directly on tumor cells and promote oncogenic processes, but also creates an immunosuppressive environment and reduce both T cell and antigen-presenting cell functions [23, 35, 36]. In particular, CD39^+^ Treg may be most prone to suppress Th17 cells [37, 38]. CD39^+^ Treg from cancer patients also suppress transendothelial migration of conventional effector T cells [39]. Indeed, CD39^+^ Treg have a superior suppressive ability compared to CD39^−^ Treg both in vivo and in vitro [24–26]. This effect is not necessarily dependent on only CD39 activity and generation of adenosine, as previous studies suggest that CD39^+^ Treg in colon tumors display several additional markers conferring suppressive ability [17, 18, 39–41]. We thus used CD39 to mark the enriched subsets of putative eTreg in our subsequent analyses of tumor-infiltrating Treg. After dividing the colon cancer patients into two groups based on the frequency of CD39^+^ Treg among intratumoral Treg, we could also show that CD39^+^ Treg in the tumors have higher Foxp3 expression on a per cell basis if they are isolated from tumors with a high infiltration of CD39^+^ Treg compared to tumors with fewer CD39^+^ Treg. This suggests that the former patients may exhibit a more tightly regulated, and less favorable, anti-tumor immune response compared to patients with fewer CD39^+^ Treg and that CD39 is a good marker for activated eTreg.

The accumulation of CD39^+^ Treg in colon tumors may result from specific recruitment, intratumoral proliferation, improved retention and upregulation of CD39 in the tumor microenvironment. Genetic polymorphisms in the *ENTPD1* gene encoding CD39 may also be an important determinant of this process. Three different single nucleotide polymorphisms (SNPs) in this gene have been reported to correlate with different levels of CD39 expression among Treg in peripheral blood of healthy donors [26] and cancer patients [17]. We observed a strong correlation between CD39^+^ Treg frequencies in the different tissues examined, indicating that *ENTPD1* SNPs may affect CD39 expression in all tissues. In particular, the correlation between CD39^+^ Treg frequencies in peripheral blood and tumor is of potential interest and indicates that CD39^+^ Treg frequencies above approximately 60% of total Treg in peripheral blood will likely correspond to patients with a large accumulation of intratumoral CD39^+^ Treg. Taken together, this suggests that frequencies of CD39^+^ Treg in peripheral blood, as well as the *ENTPD1* SNPs of each patient may serve as biomarkers to assess the corresponding CD39^+^ Treg frequencies in colon tumors.

A correlation between Treg accumulation in colorectal tumors and a poor patient survival has been demonstrated in recent studies [15, 16]. However, this correlation may be weaker for colorectal cancer compared to other solid cancer forms, based on several earlier studies showing a favorable effect of Treg on patient outcome [10, 12–14]. As most of these studies relied only on Foxp3 expression to define Treg, it is possible that activated conventional effector T cells were also included in the analysis [15]. As flow cytometry allows a much more detailed delineation of cell subsets, it is a more adequate method to identify Treg. Here, we did a further subdivision, and analysed if the accumulation of eTreg, defined by CD39, would correlate to established clinical parameters.

It should be noted that we analysed stage I to III patients with no metastases at surgery and complete removal of the tumor, a patient group that has a relatively good prognosis. This is a limitation of our study, as is the relatively low number of individuals included. Still, the evaluation of patient outcome shows a tendency towards lower cancer-specific survival in the patients with a larger proportion of intratumoral eCD39^+^ Treg. It is worth considering that there were no differences in total Treg frequencies in the tumors between patients with high and low frequencies of CD39-expressing Treg, and the potential effect seen here is not due to the presence of Treg as such, but rather the presence of an activated effector subset. The relatively low number of events registered in terms of relapse and cancer-specific death does not allow the detection of any significant differences between groups, and multivariate analysis can not be performed at this stage. Generally, patients with MSI-H tumors have a more favorable outcome than patients with MSS tumors do. In our study, patients with high and low CD39^+^ Treg frequencies had an equal distribution of MSS- and MSI-tumors. Furthermore, the other clinical parameters with a direct correlation to patient outcome that were examined did not seem to influence intratumoral CD39^+^ Treg frequencies. Thus, CD39^+^ Treg frequency in colon tumors is probably independent of the most common clinical parameters associated with patient prognosis in colon cancer. As these parameters do not co-vary with frequencies of CD39^+^ Treg, we believe that CD39 expression on Treg may be an independent variable contributing to patient outcome.

Patients with MSI-H colon tumors respond better to anti-PD-1/PD-L1 treatment compared to patients with MSS tumors [7]. PD-1 targeted treatment primarily improves effector T cell function, which is regulated in part by PD-L1 and PD-L2 expression on several different cell types, and the role of Treg suppression in this respect is not well known at present. If targeting of specific Treg subsets in colorectal cancer patients may help patients with MSS tumors, for example due to the inhibitory effect of Treg on the migration of effector T cells into tumors [39], remains to be elucidated. However, this may be an important alternative to already established cancer immunotherapies.

## Conclusions

This study presents data suggesting an association between a large intratumoral CD39^+^ Treg accumulation in colon tumors with a less favorable patient outcome. This is one step towards establishing improved treatment options for colon cancer patients, beyond PD-1 blocking, which may be especially important for patients with MSS colon tumors. Pre-clinical studies evaluating CD39-targeted therapy have shown success in the last few years [42–44], and may be a viable option for immunotherapy in MSS colon tumors with a large Treg infiltration.

## Methods

### Patients and collection of specimen

The study was performed at Sahlgrenska University Hospital and the Sahlgrenska Academy at the University of Gothenburg, in Gothenburg, Sweden. The study was approved by the Regional Research Ethics Committee of West Sweden, and all methods were performed in accordance with the relevant guidelines and regulations. Peripheral blood, unaffected colon tissue, and tumor were collected from 8 consecutive colon adenocarcinoma patients undergoing curative hemicolectomy. In a second set of experiments, material was collected from 49 additional patients, and of these, 44 patients were eligible for survival analysis and included in the study. None of the patients had undergone radiotherapy or chemotherapy for at least 3 years prior to surgery, and none suffered from autoimmune disease. Information about tumor stage was retrieved from the pathology report and medical records. Patient details and tumor characteristics are presented in Additional file 1: Table S1 and S2.

### MSS/MSI-analysis

The microsatellite status of each colon tumor was analyzed through detection and qualitative assessment of amplified and fluorescently labeled microsatellite markers using the MSI Analysis System, Version 1.2 (ProMega) as previously described [45]. In total seven markers were co-amplified using PCR-based technique, including five mononucleotide repeat markers (BAT-25, BAT-26, NR-21, NR-24 and MONO-27) and two pentanucleotide repeat markers (Penta C and Penta D). Upon completion of the PCR-part of the assay, co-amplicons were detected on the ABI PRISM® 3730 using PowerPlex 4C Matrix Standard, according to the manufacturer’s instructions. Microsatellite instability, defined as peak alterations in the dinucleotide repeats of each microsatellite marker, was assessed using the marker electropherogram of the tumor compared to corresponding unaffected colon tissue. Tumors were defined as MSI-H tumors (if > 1 of the 5 markers showed instability), MSI-L tumors (if only 1 marker showed instability) or MSS tumors (if no MSI was detected), and were analyzed using Peak scanner Software 2.

### Isolation of lymphocytes

Blood from patients was collected in heparinized tubes, and peripheral blood mononuclear cells were isolated by Ficoll-Paque (Pharmacia) density-gradient centrifugation. A biopsy of the tumor and macroscopically normal appearing mucosa from at least 10 cm away from the tumor (called unaffected colon mucosa) was collected at the time of surgery. The tissue was immediately placed in ice-cold PBS, and iEL and LPL were isolated by enzymatic digestion after removal of the epithelial fraction, essentially as described [46], but using 36 μg/ml of Liberase TL (Roche) for 1 h. For mass cytometry, CD45 leukocytes were isolated from the resulting single cell suspensions using the REAlease CD45 (TIL) MicroBead Kit (Miltenyi Biotec) according to the manufacturer’s instructions, and kept over night at 37 °C before staining and analysis. For flow cytometry analyses, isolated LPL cells were kept overnight at 4 °C and then stained with selected antibody-fluorochrome conjugates.

### Mass cytometry staining and data acquisition

We used CD45 live cell barcoding with three Platinums and 89Y to simultaneously analyse four different cell suspensions, iEL and LPL from both tumor and unaffected tissue. The different samples were individually incubated with the CD45 tags for 20 min at room temperature. After washing, cells were pooled and incubated with Human TruStain FcX (Biolegend) for 5 min at room temperature, followed by addition of the primary antibody mix for additional 30 min. See Additional file 1: Table S3 for the antibodies used. Cells were washed and stained with a secondary antibody mix consisting of anti-PE, anti-APC and anti-FITC antibodies. Subsequently, live/dead staining was performed with pre-warmed (37 °C) Rhodium-103 (Fluidigm) for 15 min at room temperature. Cells were fixed and permeabilized with the FoxP3 staining kit (eBioscience) according to manufacturer’s protocol, and FoxP3 and Ki67 were stained in Perm buffer from the staining kit for 45 min. Cells were then fixed with fresh 2% PFA for 10 min at RT, pelleted and stored in a small volume of the same buffer over night at 4 °C. Next day, samples were stained with Cell-ID Ir (Fluidigm) for 10 min, washed, and prepared for acquisition in cell acquisition solution (Fluidigm) on a Helios mass cytometer (Fluidigm).

### Mass cytometry data analyses

Mass cytometry data was analysed with OMIQ (https://www.omiq.ai/), and the heatmap in Fig. 1 was generated by using the R heatmap2 package. The data was cleaned up and manually gated as shown in Additional file 1: Figure S1. The sub-gated FoxP3^+^CD25^high^CD27^low^ Treg were exported to new FCS files with original scaling and all markers, except the ones excluded for clustering. Exported files were loaded into OMIQ and arcsinh scaling was performed (factor 5, Min -5 and Max 12,000). UMAP [47] was run with the following parameters: neighbors 15; minimum distance 0.2; components 2; metric Euclidean; learning rate 1; epochs 400; random seed 2390; embedding initialization spectral. Phenograph [48] was run with the following parameters: k 20; distance metric Euclidean, Louvain seed 7617, Louvain runs 1, number of results 1. Phenograph computed 18 clusters of which 5 clusters contained events less than 1% of all Treg in both tumors and unaffected tissues. These were excluded from further analysis.

### Flow cytometry analyses

Flow cytometry analyses were performed on single cells after excluding dead cells with Live/Dead fixable aqua dead cell stain kit (Molecular Probes). Data acquisition was performed on a LSRII flow cytometer or a FACS Aria (BD Biosciences), equipped with FACS Diva software (BD Biosciences) and analyzed using FlowJo software (TreeStar Inc). The antibodies used are listed in Additional file 1: Table S4. To stain for Foxp3 we used the Foxp3 Staining Buffer Set (eBioscience) for intracellular staining, and Treg were identified as CD3^+^CD4^+^FoxP3^+^CD25^high^ cells within the lymphocyte gate (see Additional file 1: Figure S5) [49]. Isotype controls were used as reference to determine cut-off levels for specific expression.

### Survival analysis

Of the 49 colon cancer patients selected to participate in the study, five patients were not admissible for survival analysis and thus excluded from the study; two patients due to non-radical surgery; two patients due to a non-primary tumor; and one patient due to moving abroad which made follow-up impossible. Of the remaining 44 patients included in the survival analysis eight patients were censored due to non-cancer related death but remained in the study until they were censored. The time to event ranged from 81 to 1071 days (in relation to relapse), 372–734 days (in relation to cancer-specific death), and the time to censorship ranged from 109 days to the end of the study (also including censorship from non-cancer specific death). The median follow-up time was 1524 days for patients that had been censored based on follow-up time. Estimated survivor curves were performed using the Kaplan–Meier method and the SPSS program.

### Statistical methods

Statistical analyses were performed with Prism (GraphPad, US), using Wilcoxon matched-pairs signed rank test to compare paired data from the same patient, and Mann–Whitney and Kruskal–Wallis tests to compare data from different patients, comprising two groups and more than two groups, respectively. Spearman correlation tests were used for correlation analyses, and Log-Rank test for comparison of Kaplan–Meier curves. *p* values of < 0.02 were considered significant.

## Supplementary Information

**Additional file1: Table S1.** Characteristics of the colon cancer patients included in mass cytometry analyses. **Table S2.** Characteristics of the colon cancer patients included in the survival analysis. **Table S3.** List of antibodies used in mass cytometry experiments. **Table S4.** List of antibodies used in flow cytometry experiments. **Figure S1.** Gating strategy for mass cytometry experiments. **Figure S2.** Color overlay of all protein markers used in Fig. 1 on the UMAP plot. **Figure S3.** Expression of ICOS, OX-40, CD39, and CD56 in the different Phenograph clusters. **Figure S4.** Paired analysis of Phenograph cluster frequencies. **Figure S5.** Gating strategy for identification of Treg. **Figure S6.** Correlation between CD39 expression by Treg in different tissues.

## Data Availability

The datasets analysed during the current study are available from the corresponding author on reasonable request.

## Abbreviations

CRC: Colorectal cancer
ENTPD1: Ectonucleoside triphosphate diphosphohydrolase-1
eTreg: Effector Treg
ICOS: Inducible T-cell co-stimulator
LAG-3: Lymphocyte activation gene 3
LPL: Lamina propria lymphocytes
MFI: Mean fluorescence intensity
MSI-H: Microsatellite instability high
MSI-L: Microsatellite instability low
MSS: Microsatellite stable
PD-1: Programmed cell death protein 1
PD-L1: Programmed death-ligand 1
PD-L2: Programmed death-ligand 2
SNP: Single nucleotide polymorphism
TIGIT: T cell immunoreceptor with Ig and ITIM domains
Treg: Regulatory T cell
UMAP: Uniform Manifold Approximation and Projection

## References

[1] Fridman WH, Mlecnik B, Bindea G, Pages F, Galon J (2011). Immunosurveillance in human non-viral cancers. Curr Opin Immunol.

[2] Brenner H, Kloor M, Pox CP (2014). Colorectal cancer. Lancet.

[3] Bindea G, Mlecnik B, Tosolini M, Kirilovsky A, Waldner M, Obenauf AC, Angell H, Fredriksen T, Lafontaine L, Berger A (2013). Spatiotemporal dynamics of intratumoral immune cells reveal the immune landscape in human cancer. Immunity.

[4] Tosolini M, Kirilovsky A, Mlecnik B, Fredriksen T, Mauger S, Bindea G, Berger A, Bruneval P, Fridman WH, Pages F (2011). Clinical impact of different classes of infiltrating T cytotoxic and helper cells (Th1, th2, treg, th17) in patients with colorectal cancer. Cancer Res.

[5] Mlecnik B, Bindea G, Angell HK, Maby P, Angelova M, Tougeron D, Church SE, Lafontaine L, Fischer M, Fredriksen T (2016). Integrative analyses of colorectal cancer show immunoscore is a stronger predictor of patient survival than microsatellite instability. Immunity.

[6] Cogdill AP, Andrews MC, Wargo JA (2017). Hallmarks of response to immune checkpoint blockade. Br J Cancer.

[7] Le DT, Uram JN, Wang H, Bartlett BR, Kemberling H, Eyring AD, Skora AD, Luber BS, Azad NS, Laheru D (2015). PD-1 blockade in tumors with mismatch-repair deficiency. N Engl J Med.

[8] Germano G, Amirouchene-Angelozzi N, Rospo G, Bardelli A (2018). The clinical impact of the genomic landscape of mismatch repair-deficient cancers. Cancer Discov.

[9] Llosa NJ, Cruise M, Tam A, Wicks EC, Hechenbleikner EM, Taube JM, Blosser RL, Fan H, Wang H, Luber BS (2015). The vigorous immune microenvironment of microsatellite instable colon cancer is balanced by multiple counter-inhibitory checkpoints. Cancer Discov.

[10] Chen X, Du Y, Lin X, Qian Y, Zhou T, Huang Z (2016). CD4+CD25+ regulatory T cells in tumor immunity. Int Immunopharmacol.

[11] deLeeuw RJ, Kost SE, Kakal JA, Nelson BH (2012). The prognostic value of FoxP3+ tumor-infiltrating lymphocytes in cancer: a critical review of the literature. Clin Cancer Res.

[12] Salama P, Phillips M, Grieu F, Morris M, Zeps N, Joseph D, Platell C, Iacopetta B (2009). Tumor-infiltrating FOXP3+ T regulatory cells show strong prognostic significance in colorectal cancer. J Clin Oncol.

[13] Frey DM, Droeser RA, Viehl CT, Zlobec I, Lugli A, Zingg U, Oertli D, Kettelhack C, Terracciano L, Tornillo L (2010). High frequency of tumor-infiltrating FOXP3(+) regulatory T cells predicts improved survival in mismatch repair-proficient colorectal cancer patients. Int J Cancer.

[14] Ling A, Edin S, Wikberg ML, Oberg A, Palmqvist R (2014). The intratumoural subsite and relation of CD8(+) and FOXP3(+) T lymphocytes in colorectal cancer provide important prognostic clues. Br J Cancer.

[15] Saito T, Nishikawa H, Wada H, Nagano Y, Sugiyama D, Atarashi K, Maeda Y, Hamaguchi M, Ohkura N, Sato E (2016). Two FOXP3(+)CD4(+) T cell subpopulations distinctly control the prognosis of colorectal cancers. Nat Med.

[16] De Simone M, Arrigoni A, Rossetti G, Gruarin P, Ranzani V, Politano C, Bonnal RJP, Provasi E, Sarnicola ML, Panzeri I (2016). Transcriptional landscape of human tissue lymphocytes unveils uniqueness of tumor-infiltrating T regulatory cells. Immunity.

[17] Timperi E, Pacella I, Schinzari V, Focaccetti C, Sacco L, Farelli F, Caronna R, Del Bene G, Longo F, Ciardi A (2016). Regulatory T cells with multiple suppressive and potentially pro-tumor activities accumulate in human colorectal cancer. Oncoimmunology.

[18] Ahlmanner F, Sundstrom P, Akeus P, Eklof J, Borjesson L, Gustavsson B, Lindskog EB, Raghavan S, Quiding-Jarbrink M (2018). CD39(+) regulatory T cells accumulate in colon adenocarcinomas and display markers of increased suppressive function. Oncotarget.

[19] Scurr M, Ladell K, Besneux M, Christian A, Hockey T, Smart K, Bridgeman H, Hargest R, Phillips S, Davies M (2014). Highly prevalent colorectal cancer-infiltrating LAP(+) Foxp3(-) T cells exhibit more potent immunosuppressive activity than Foxp3(+) regulatory T cells. Mucosal Immunol.

[20] Tanaka A, Sakaguchi S (2017). Regulatory T cells in cancer immunotherapy. Cell Res.

[21] Michel S, Benner A, Tariverdian M, Wentzensen N, Hoefler P, Pommerencke T, Grabe N, von Knebel DM, Kloor M (2008). High density of FOXP3-positive T cells infiltrating colorectal cancers with microsatellite instability. Br J Cancer.

[22] Svensson H, Olofsson V, Lundin S, Yakkala C, Bjorck S, Borjesson L, Gustavsson B, Quiding-Jarbrink M (2012). Accumulation of CCR4(+)CTLA-4 FOXP3(+)CD25(hi) regulatory T cells in colon adenocarcinomas correlate to reduced activation of conventional T cells. PLoS ONE.

[23] Stagg J, Smyth MJ (2010). Extracellular adenosine triphosphate and adenosine in cancer. Oncogene.

[24] Deaglio S, Dwyer KM, Gao W, Friedman D, Usheva A, Erat A, Chen JF, Enjyoji K, Linden J, Oukka M (2007). Adenosine generation catalyzed by CD39 and CD73 expressed on regulatory T cells mediates immune suppression. J Exp Med.

[25] Gu J, Ni X, Pan X, Lu H, Lu Y, Zhao J, Guo Zheng S, Hippen KL, Wang X, Lu L (2017). Human CD39(hi) regulatory T cells present stronger stability and function under inflammatory conditions. Cell Mol Immunol.

[26] Rissiek A, Baumann I, Cuapio A, Mautner A, Kolster M, Arck PC, Dodge-Khatami A, Mittrucker HW, Koch-Nolte F, Haag F (2015). The expression of CD39 on regulatory T cells is genetically driven and further upregulated at sites of inflammation. J Autoimmun.

[27] Miller KD, Siegel RL, Lin CC, Mariotto AB, Kramer JL, Rowland JH, Stein KD, Alteri R, Jemal A (2016). Cancer treatment and survivorship statistics, 2016. CA Cancer J Clin.

[28] Saha S, Shaik M, Johnston G, Saha SK, Berbiglia L, Hicks M, Gernand J, Grewal S, Arora M, Wiese D (2015). Tumor size predicts long-term survival in colon cancer: an analysis of the National Cancer Data Base. Am J Surg.

[29] Thibaudin M, Chaix M, Boidot R, Vegran F, Derangere V, Limagne E, Berger H, Ladoire S, Apetoh L, Ghiringhelli F (2016). Human ectonucleotidase-expressing CD25(high) Th17 cells accumulate in breast cancer tumors and exert immunosuppressive functions. Oncoimmunology.

[30] Bartolome-Casado R, Landsverk OJB, Chauhan SK, Saetre F, Hagen KT, Yaqub S, Oyen O, Horneland R, Aandahl EM, Aabakken L (2021). CD4(+) T cells persist for years in the human small intestine and display a TH1 cytokine profile. Mucosal Immunol.

[31] Mitchell J, Kelly J, Kvedaraite E, von Bahr GT, Henter JI, Pellicci DG, Berzins SP, Kannourakis G (2020). Foxp3(+) Tregs from Langerhans cell histiocytosis lesions co-express CD56 and have a definitively regulatory capacity. Clin Immunol.

[32] Li X, Peng J, Pang Y, Yu S, Yu X, Chen P, Wang W, Han W, Zhang J, Yin Y (2015). Identification of a FOXP3(+)CD3(+)CD56(+) population with immunosuppressive function in cancer tissues of human hepatocellular carcinoma. Sci Rep.

[33] Whiteside TL, Mandapathil M, Schuler P (2011). The role of the adenosinergic pathway in immunosuppression mediated by human regulatory T cells (Treg). Curr Med Chem.

[34] Mandapathil M, Hilldorfer B, Szczepanski MJ, Czystowska M, Szajnik M, Ren J, Lang S, Jackson EK, Gorelik E, Whiteside TL (2010). Generation and accumulation of immunosuppressive adenosine by human CD4+CD25highFOXP3+ regulatory T cells. J Biol Chem.

[35] Moesta AK, Li XY, Smyth MJ (2020). Targeting CD39 in cancer. Nat Rev Immunol.

[36] Allard B, Longhi MS, Robson SC, Stagg J (2017). The ectonucleotidases CD39 and CD73: novel checkpoint inhibitor targets. Immunol Rev.

[37] Fletcher JM, Lonergan R, Costelloe L, Kinsella K, Moran B, O'Farrelly C, Tubridy N, Mills KH (2009). CD39+Foxp3+ regulatory T Cells suppress pathogenic Th17 cells and are impaired in multiple sclerosis. J Immunol.

[38] Grant CR, Liberal R, Holder BS, Cardone J, Ma Y, Robson SC, Mieli-Vergani G, Vergani D, Longhi MS (2014). Dysfunctional CD39(POS) regulatory T cells and aberrant control of T-helper type 17 cells in autoimmune hepatitis. Hepatology.

[39] Sundstrom P, Stenstad H, Langenes V, Ahlmanner F, Theander L, Ndah TG, Fredin K, Borjesson L, Gustavsson B, Bastid J (2016). Regulatory T cells from colon cancer patients inhibit effector T-cell migration through an adenosine-dependent mechanism. Cancer Immunol Res.

[40] Syed Khaja AS, Toor SM, El Salhat H, Ali BR, Elkord E (2017). Intratumoral FoxP3(+)Helios(+) regulatory T cells upregulating immunosuppressive molecules are expanded in human colorectal cancer. Front Immunol.

[41] Strasser K, Birnleitner H, Beer A, Pils D, Gerner MC, Schmetterer KG, Bachleitner-Hofmann T, Stift A, Bergmann M, Oehler R (2019). Immunological differences between colorectal cancer and normal mucosa uncover a prognostically relevant immune cell profile. Oncoimmunology.

[42] Bastid J, Regairaz A, Bonnefoy N, Dejou C, Giustiniani J, Laheurte C, Cochaud S, Laprevotte E, Funck-Brentano E, Hemon P (2015). Inhibition of CD39 enzymatic function at the surface of tumor cells alleviates their immunosuppressive activity. Cancer Immunol Res.

[43] Li XY, Moesta AK, Xiao C, Nakamura K, Casey M, Zhang H, Madore J, Lepletier A, Aguilera AR, Sundarrajan A (2019). Targeting CD39 in cancer reveals an extracellular ATP- and inflammasome-driven tumor immunity. Cancer Discov.

[44] Perrot I, Michaud HA, Giraudon-Paoli M, Augier S, Docquier A, Gros L, Courtois R, Dejou C, Jecko D, Becquart O (2019). Blocking antibodies targeting the CD39/CD73 immunosuppressive pathway unleash immune responses in combination cancer therapies. Cell Rep.

[45] Sundstrom P, Szeponik L, Ahlmanner F, Sundquist M, Wong JSB, Lindskog EB, Gustafsson B, Quiding-Jarbrink M (2019). Tumor-infiltrating mucosal-associated invariant T (MAIT) cells retain expression of cytotoxic effector molecules. Oncotarget.

[46] Lundgren A, Stromberg E, Sjoling A, Lindholm C, Enarsson K, Edebo A, Johnsson E, Suri-Payer E, Larsson P, Rudin A (2005). Mucosal FOXP3-expressing CD4+ CD25high regulatory T cells in Helicobacter pylori-infected patients. Infect Immun.

[47] McInnes L, Healy J, Melville J (2018). UMAP: uniform manifold approximation and projection for dimension reduction. I open Source Softw.

[48] Levine JH, Simonds EF, Bendall SC, Davis KL, el Amir AD, Tadmor MD, Litvin O, Fienberg HG, Jager A, Zunder ER (2015). Data-driven phenotypic dissection of AML reveals progenitor-like cells that correlate with prognosis. Cell.

[49] Santegoets SJ, Dijkgraaf EM, Battaglia A, Beckhove P, Britten CM, Gallimore A, Godkin A, Gouttefangeas C, de Gruijl TD, Koenen HJ (2015). Monitoring regulatory T cells in clinical samples: consensus on an essential marker set and gating strategy for regulatory T cell analysis by flow cytometry. Cancer Immunol Immunother.

