# Supplementary material for: Intratumoral regulatory T cells from colon cancer patients comprise several activated effector populations
Source: BMC Immunol. 2021 Aug 19;22:58. doi: 10.1186/s12865-021-00449-1 (PMC8375143; doi:10.1186/s12865-021-00449-1)
Supplement: Supplementary file 1 — Additional file1: Table S1. Characteristics of the colon cancer patients included in mass cytometry analyses. Table S2. Characteristics of the colon cancer patients included in the survival analysis. Table S3. List of antibodies used in mass cytometry experiments. Table S4. List of antibodies used in flow cytometry experiments. Figure S1. Gating strategy for mass cytometry experiments. Figure S2. Color overlay of all protein markers used in Fig. 1 on the UMAP plot. Figure S3. Expression of ICOS, OX-40, CD39, and CD56 in the different Phenograph clusters. Figure S4. Paired analysis of Phenograph cluster frequencies. Figure S5. Gating strategy for identification of Treg. Figure S6. Correlation between CD39 expression by Treg in different tissues. [file 12865_2021_449_MOESM1_ESM.pdf]

Supplementary table S1. Characteristics of the colon cancer patients included in mass cytometry analyses.

ID	age	sex	tumor location	TNM stage	microsatellite status
433	75	M	caekum	T3N0M0	MSS
435	80	F	caekum	T2N0M0	MSS
441	44	M	transverse colon	T3N1M0	MSS
442	83	M	sigmoid colon	T3N2M0	MSS
443	57	M	descending colon	T3N1M0	MSS
444	94	M	caekum	T3N1M0	MSS
445	60	M	sigmoid colon	T4N0M1	MSS
448	88	M	transverse colon	T2N0M0	MSS

Supplementary Table S2. Characteristics of the colon cancer patients included in the survival analysis.

		females	males
	n	22	22
	age	62-87	46-93
Tumor location	right colon	20	12
	left colon	2	9
	several locations	-	1
stage	I	2	2
	lI	10	9
	III	10	11
	IV	-	-
Microsatellite status	MSS	14	20
	MSI-H	8	2

Supplementary Table S3. List of antibodies used in mass cytometry experiments

metal/fluorescence tag	target	clone	manufacturer
143Nd	CD352 (NTB-A)	NT-7	Fluidigm
144Nd	CD69	FN50	Fluidigm
148Nd	CD278/ICOS	C398.4A	Fluidigm
149Sm	CD127 (IL-7Ra)	A019D5	Fluidigm
150Nd	CD223/LAG-3	11C3C65	Fluidigm
151Eu	CD103	Ber-ACT8	Fluidigm
152Sm	TCRγδ	11F2	Fluidigm
153Eu	TCR Vα7.2 (MAIT cell)	3C10	Fluidigm
155Gd	CD45RA	HI100	Fluidigm
158Gd	CD137/4-1BB	4B4-1	Fluidigm
159Tb	CD337 (NKp30)	Z25	Fluidigm
160Gd	CD28	CD28.2	Fluidigm
164Dy	CD95/Fas	DX2	Fluidigm
165Ho	CD45RO	UCHL1	Fluidigm
166Er	CD314 (NKG2D)	ON72	Fluidigm
167Er	CD158e1 (KIR3DL1, NKB1)	DX9	Fluidigm
169Tm	CD159a (NKG2A)	Z199	Fluidigm
170Er	TCR Vα24-Jα18 (iNKT cell)	6B11	Fluidigm
171Yb	CD66a/CEACAM1	CD66a-B1.1	Fluidigm
172Yb	CD38	HIT2	Fluidigm
173Yb	CD158b (KIR2DL2/L3, NKAT2)	DX27	Fluidigm
174Yb	CD94	HP-3D9	Fluidigm
175Lu	CD279 (PD-1)	EH12.2H7	Fluidigm
176Yb	CD56 (NCAM)	NCAM16.2	Fluidigm
209Bi	CD16	3G8	Fluidigm
156Gd	PE (Fluorochrome)	PE001	Fluidigm
163Dy	APC (Fluorochrome)	APC003	Fluidigm
162Dy	FoxP3	259D/C7	Fluidigm
168Er	Ki-67	B56	Fluidigm
106 Cd	CD86	IT2.2	Biolegend, self-conj.
110 Cd	Vδ2	B6	Biolegend, self-conj.
111 Cd	CD62L	DREG-56	Biolegend, self-conj.
113 Cd	CD3	SK7	Biolegend, self-conj.
116 Cd	CD8α	RPA-T8	Biolegend, self-conj.
161Dy	TIGIT	MBSA43	Biolegend, self-conj.
145Nd	CD39	A1	Biolegend, self-conj.
146Nd	CD84	CD84.1.21	Biolegend, self-conj.
147Sm	CD134 (OX-40)	ACT35	Biolegend, self-conj.
141Pr	CD25 (IL-2R)	2A3	Biolegend, self-conj.
142Nd	CD161	HP-3G10	Biolegend, self-conj.
Cd112	FITC (Fluorochrome)	FIT-22	Biolegend, self-conj.
Pt194,Pt196,Pt198	CD45	HI30	Biolegend, self-conj.

Supplementary table S4. Antibodies used for flow cytometry analyses

**marker**	**Ab clone**	**fluorochrome**	**distributer**
CD3	UCHT1	AF700	BioLegend
CD4	OKT4	PerCP	BioLegend
CD8	RPA-T8	BV711	BD Biosciences
CD25	M-A251	BV650	BD Biosciences
CD39	A1	FITC	AbD Serotec
Foxp3	PCH101	APC	eBioscience

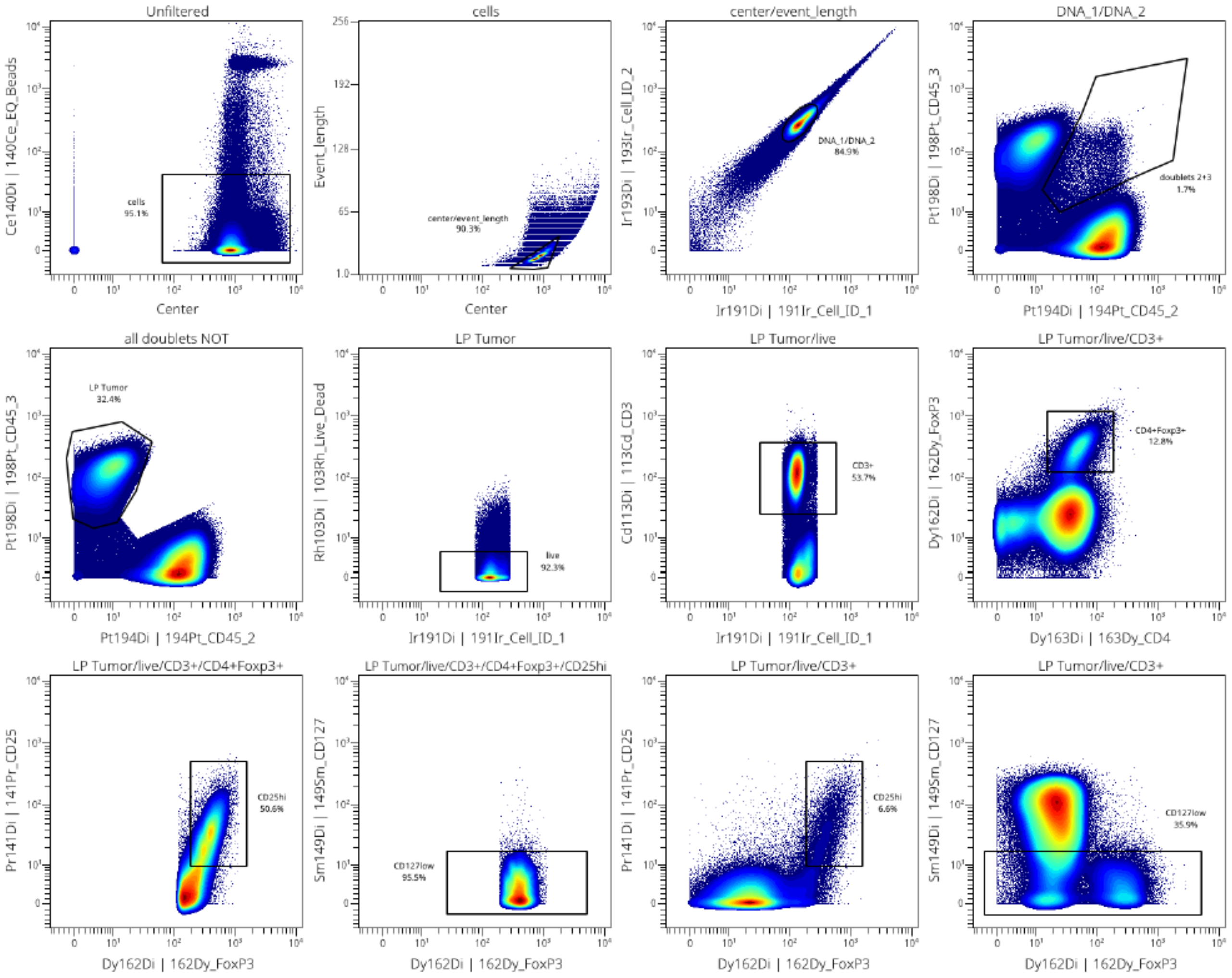

Supplementary Figure S1. Representative **g**ating strategy for mass cytometry experiments. Gating strategy shows the clean-up (doublet gating is an example of all possible combinations of the four bar codes), de-barcoding and gating of $CD3^{+}CD4^{+}Foxp3^{+}CD25^{hi}CD127^{low}$ Treg for further analysis. The last two plots show the same gates for $Foxp3^{+}CD25^{hi}$ cells and $CD127^{low}$ cells among all $CD3^{+}$ cells in a representative tumor sample for comparison purposes

Suppl. Fig. S2

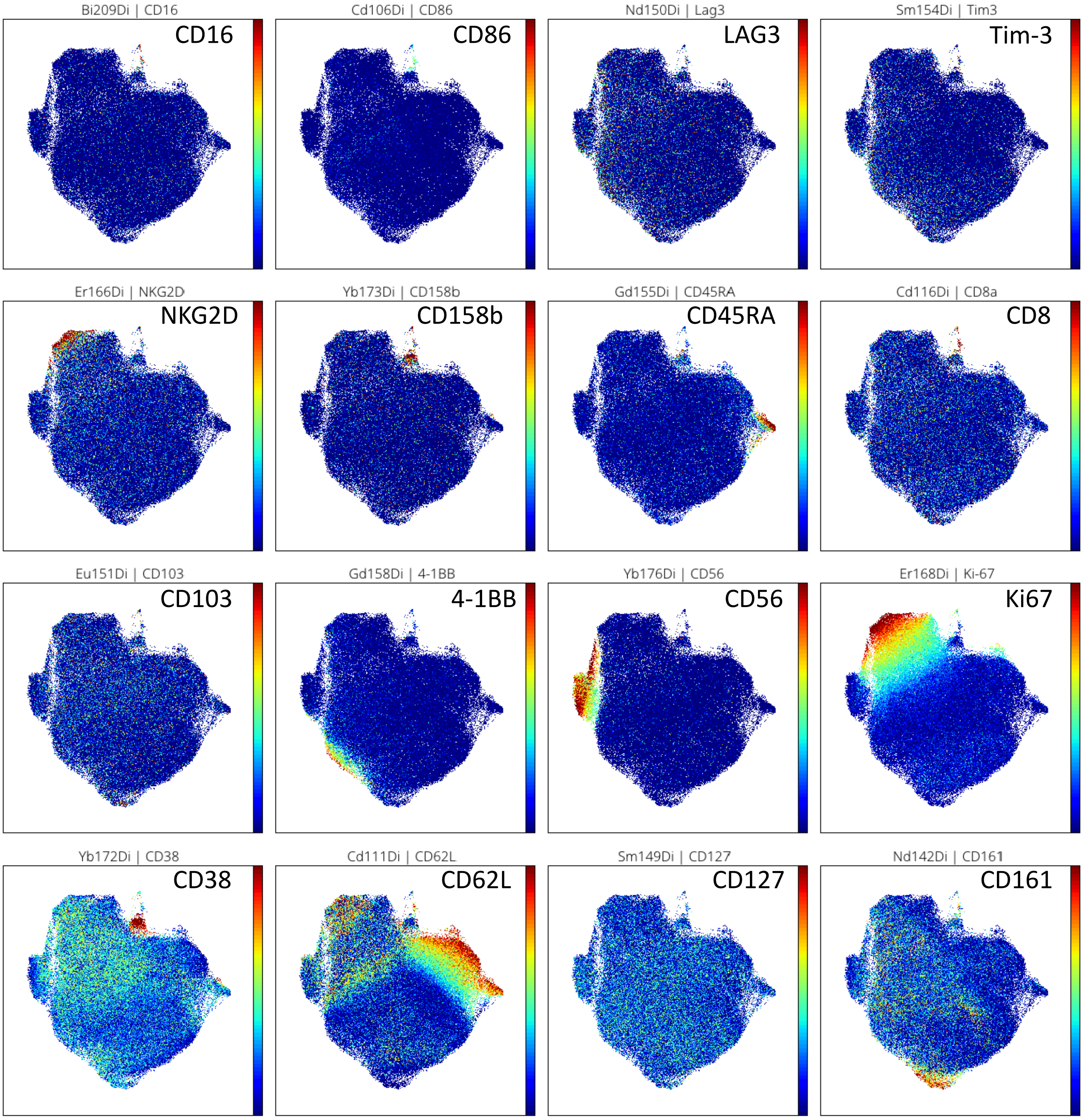

Bi209Di | CD16
CD16
Cd106Di | CD86
CD86
Nd150Di | Lag3
LAG3
Sm154Di | Tim3
Tim-3
Er166Di | NKG2D
NKG2D
Yb173Di | CD158b
CD158b
Gd155Di | CD45RA
CD45RA
Cd116Di | CD8a
CD8
Eu151Di | CD103
CD103
Gd158Di | 4-1BB
4-1BB
Yb176Di | CD56
CD56
Er168Di | Ki-67
Ki67
Yb172Di | CD38
CD38
Cd111Di | CD62L
CD62L
Sm149Di | CD127
CD127
Nd142Di | CD161
CD161

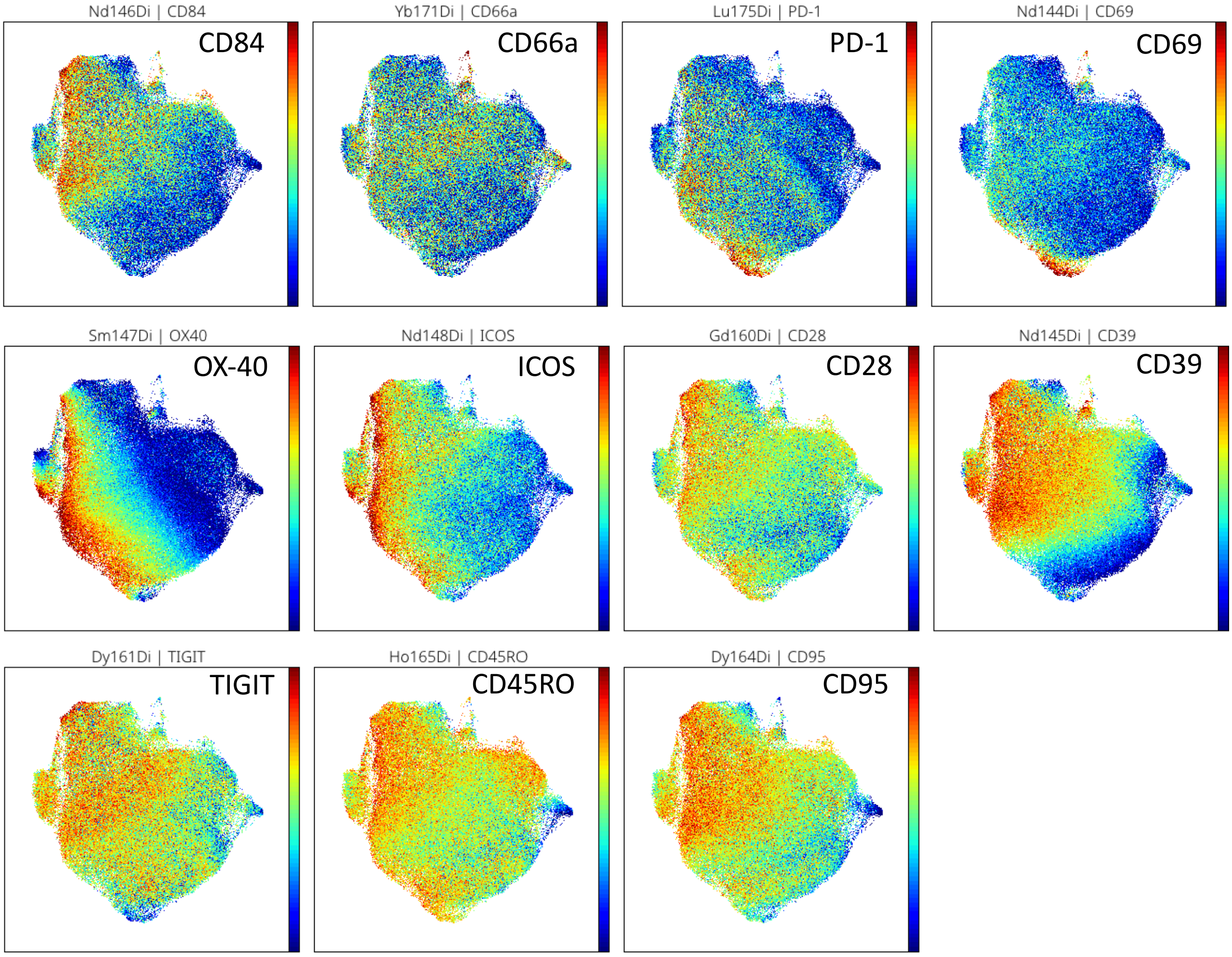

Supplementary Figure S2. Expression of individual molecules (indicated in the upper right corner of each panel) is visualized on the UMAP plot. Colour shows intesity of marker expression going from blue (low) to red (high).

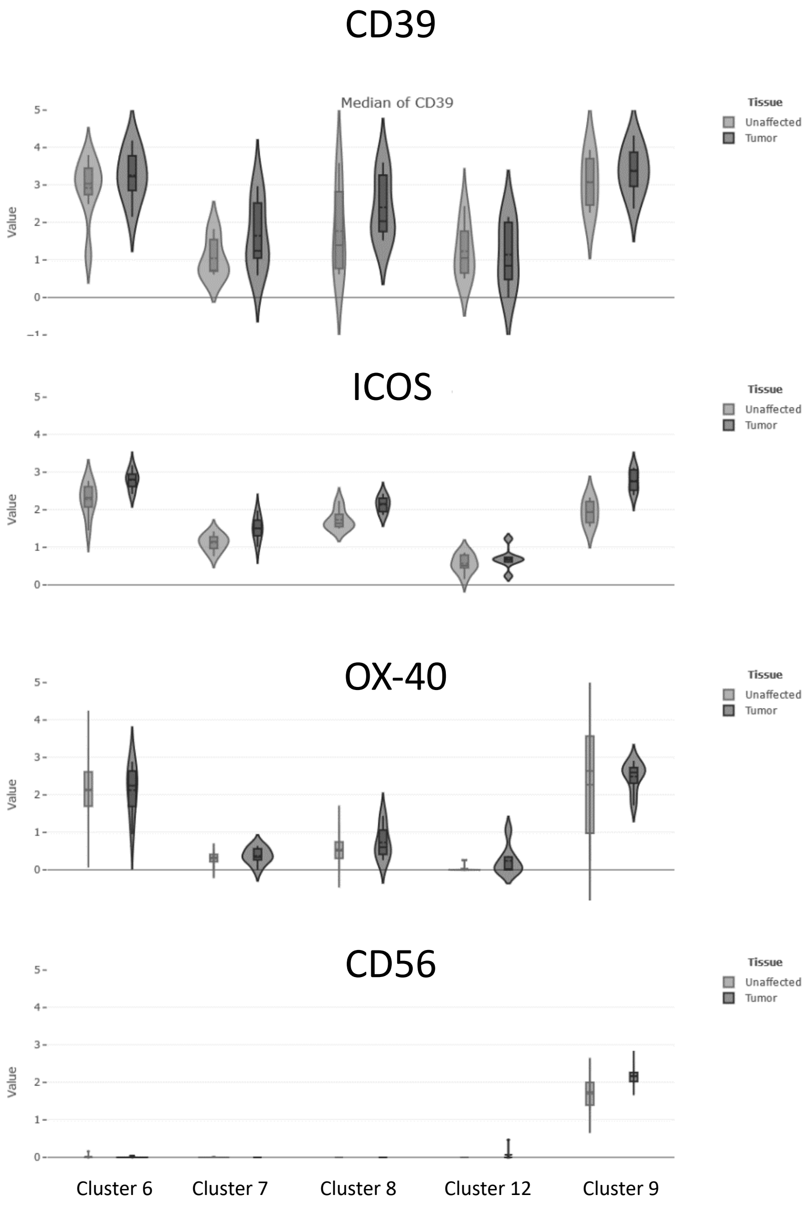

Supplementary Figure S3. Violin plots of median expression of CD39, ICOS, OX-40 and CD56 for Phenograph clusters in unaffected and tumor tissue. Shown are clusters with a p-value >0.02 (Wilcoxon test). Cluster 9 does not contain any cells in 4 (of 8) samples in the unaffected tissue and therefore shows a large spread in the violin plot.

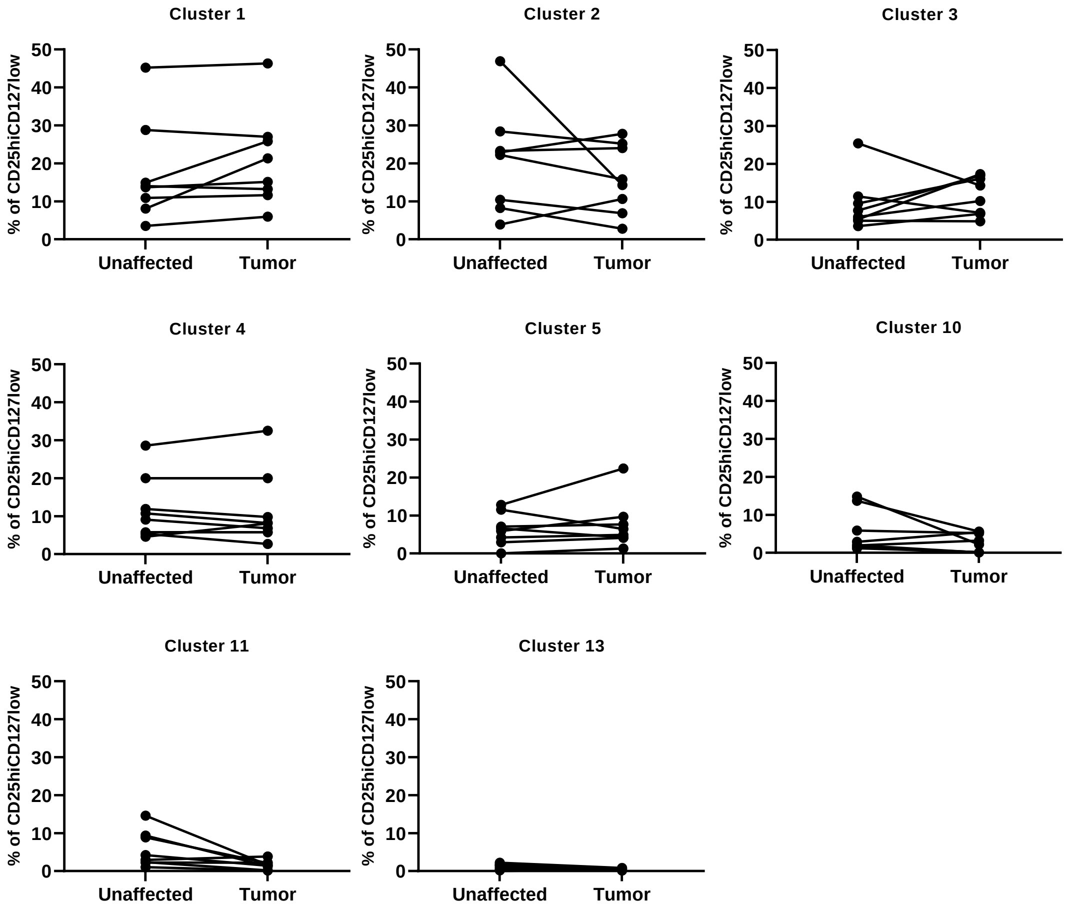

Supplementary Figure S4. Paired analysis of Phenograph cluster frequencies of FoxP3^{+}CD25^{hi}CD127^{low} Treg in unaffected and tumor tissue. Symbols represent individual values and unafected tissue and tumor from the same patients are connected. Clusters with a p value >0.02 (Wilcoxon test) are shown.

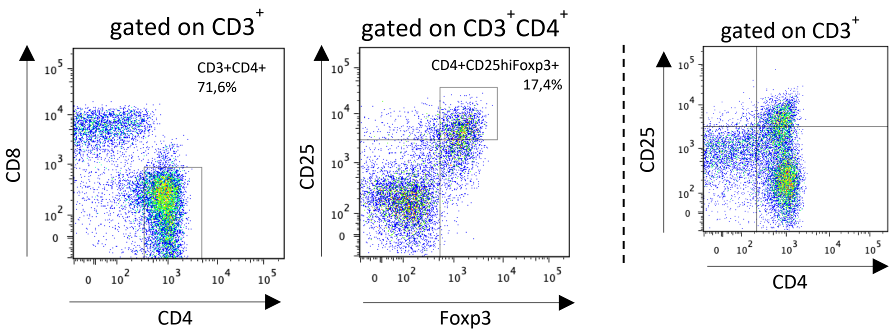

Supplentary Figure S5. Gating strategy for identification of Treg. Single cell suspensions were isolated from blood and colon tissues, stained and analyzed by flow cytometry. The dot-plots illustrate the gating strategy to identify $CD3^{+}CD4^{+}CD25^{hi}Foxp3^{+}$ Treg in a colon tumor sample. Foxp3 expression was determined using an isotype control and a dot-plot to aid for gate placement (to the far right) was used to identify $CD25^{high}$ cells.

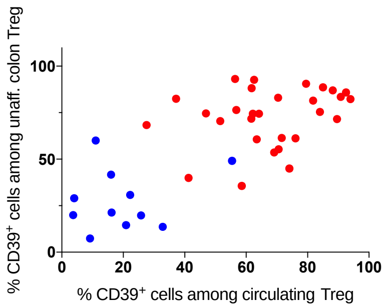

Spearman: r = 0,65; p <0.0001

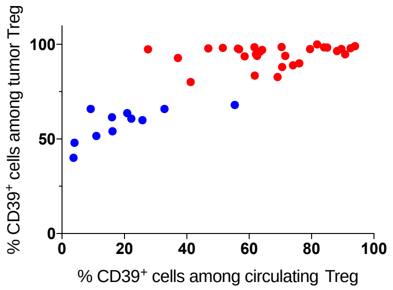

Spearman: r = 0,70; p <0.0001

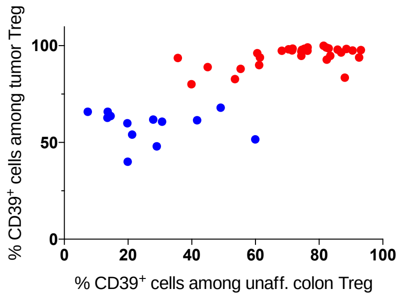

Spearman: r = 0,75; p <0.0001

Supplementary Figure S6. Correlation between CD39 expression by Treg in different tissues. Colon tumors were divided into group I and II tumors based on CD39 expression by isolated intratumoral Treg in flow cytometry analysis. Correlation between frequencies of $CD39^{+}$ Treg among total Treg in peripheral blood, unaffected colon tissue and tumor tissue was analyzed using Spearman rank correlation. Symbols represent individual values (n = 40–43) and data is color-coded in red (group I) and blue (group II).
